# Poly[dibutyl­ammonium [nonamethylbis­(μ_3_-sulfato-κ^3^ *O*:*O*′:*O*′′)tristannate(IV)]]

**DOI:** 10.1107/S1600536812042894

**Published:** 2012-10-20

**Authors:** Tidiane Diop, Libasse Diop, Arie van Lee

**Affiliations:** a Laboratoire de Chimie Minérale et Analytique, Département de Chimie, Faculté des Sciences et Techniques, Université Cheikh Anta Diop, Dakar, Senegal; b Institut Europeen des Membranes, Universite de Montpellier II, 34000 Montpellier, France

## Abstract

In the structure of the title coordination polymer, {(C_8_H_20_N)[Sn_3_(CH_3_)_9_(SO_4_)_2_]}_*n*_, each of the three Sn^IV^ atoms is coordinated in a trigonal–bipyramidal manner by three methyl groups in the equatorial plane and by two O atoms of SO_4_^2−^ anions in the axial positions. The μ_3_-bridging mode of the sulfate anions leads to the formation of corrugated anionic layers parallel to (100). The uncoordinating O atom of one of the two SO_4_^2−^ anions is N—H⋯O hydrogen-bonded to the dibutyl­ammonium cation inter­connecting the anionic sheets. The structure is partially disordered. The dibutyl ammonium ion is found on two positions with an occupancy ratio of 0.525 (10):0.475 (10), and one sulfate group with three connecting trimethyl stannyl groups is also positionally disordered over two sets of sites with an occupancy ratio of 0.725 (4):0.275 (4).

## Related literature

For applications of tin(IV) compounds, see: Basu *et al.* (2005 ▶); Evans & Karpel (1985 ▶); Samuel *et al.* (2002 ▶); Kapoor *et al.* (2005 ▶). For related organotin(IV) compounds, see: Molloy *et al.* (1989 ▶); Diop *et al.* (2002 ▶) Diallo *et al.* (2009 ▶).
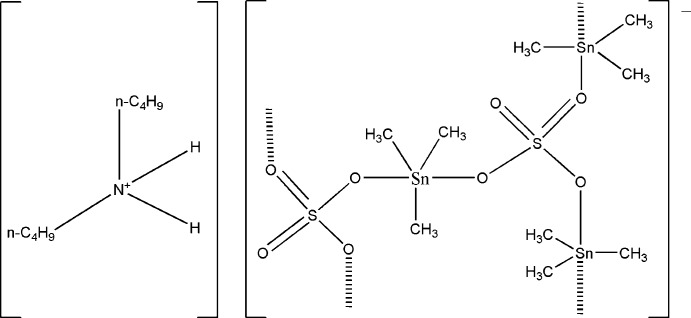


## Experimental

### 

#### Crystal data

(C_8_H_20_N)[Sn_3_(CH_3_)_9_(SO_4_)_2_]
*M* *_r_* = 813.75
Monoclinic, 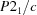

*a* = 11.8847 (2) Å
*b* = 18.2884 (3) Å
*c* = 15.3949 (2) Å
β = 107.380 (1)°
*V* = 3193.35 (9) Å^3^
*Z* = 4
Mo *K*α radiation
μ = 2.49 mm^−1^
*T* = 173 K
0.06 × 0.04 × 0.04 mm

#### Data collection

Bruker SMART CCD diffractometer
Absorption correction: multi-scan (*SADABS*, Bruker, 2003 ▶) *T*_min_ = 0.865, *T*_max_ = 0.907
69889 measured reflections
7234 independent reflections
6265 reflections with *I* > 2σ(*I*)
*R*_int_ = 0.027

#### Refinement

*R*[*F* ^2^ > 2σ(*F* ^2^)] = 0.020
*wR*(*F* ^2^) = 0.045
*S* = 1.06
7234 reflections
463 parameters
77 restraints
H-atom parameters constrained
Δρ_max_ = 0.76 e Å^−3^
Δρ_min_ = −0.46 e Å^−3^

### 

Data collection: *SMART* (Bruker, 2003 ▶); cell refinement: *SAINT* (Bruker, 2003 ▶); data reduction: *SAINT*; program(s) used to solve structure: *SHELXS97* (Sheldrick, 2008 ▶); program(s) used to refine structure: *SHELXLE* (Hübschle *et al.*, 2011 ▶); molecular graphics: OLEX2 (Dolomanov *et al.*, 2009 ▶); software used to prepare material for publication: *publCIF* (Westrip (2010 ▶).

## Supplementary Material

Crystal structure: contains datablock(s) I, New_Global_Publ_Block. DOI: 10.1107/S1600536812042894/wm2692sup1.cif

Structure factors: contains datablock(s) I. DOI: 10.1107/S1600536812042894/wm2692Isup2.hkl

Additional supplementary materials:  crystallographic information; 3D view; checkCIF report

## Figures and Tables

**Table 1:** Hydrogen-bond geometry (Å, °)

*D*—H⋯*A*	*D*—H	H⋯*A*	*D*⋯*A*	*D*—H⋯*A*
N1—H1*H*⋯O6	0.92	1.88	2.785 (16)	168
N1*B*—H1*J*⋯O6*B*	0.92	2.11	2.98 (2)	159

**Fig. 1.**
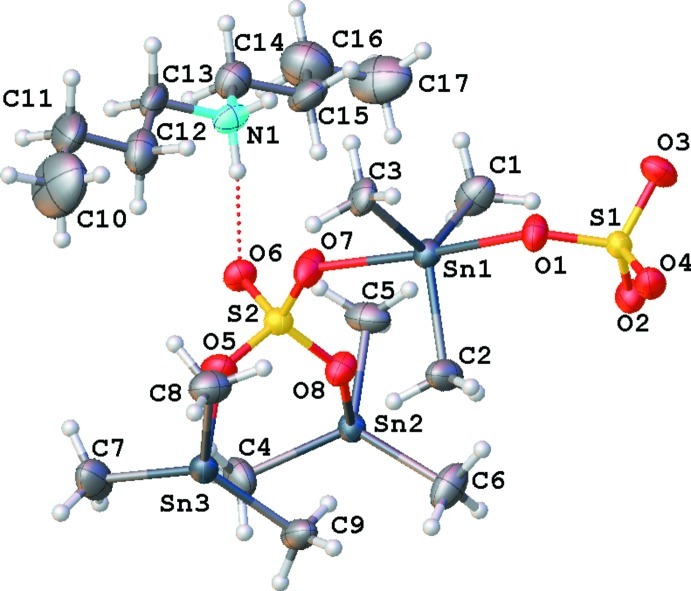
The asymmetric unit of the structure of compound (I) with displacement ellipsoids drawn at the 50° probability level. The minor components of the disordered parts within the structure are not shown.

**Fig. 2.**
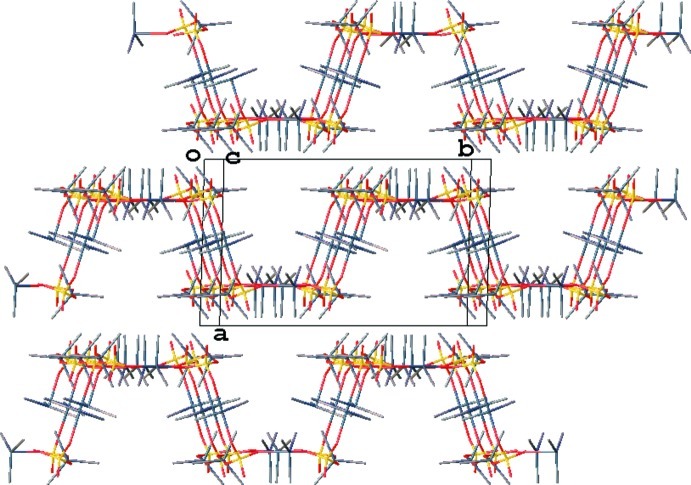
The two-dimensional [(SnMe3)3(SO4)2]- anionic sheet structure of (I). Hydrogen atoms and the Bu2NH2+ cations have been omitted for clarity. The minor components of the disordered parts within the structure are not shown.

**Fig. 3.**
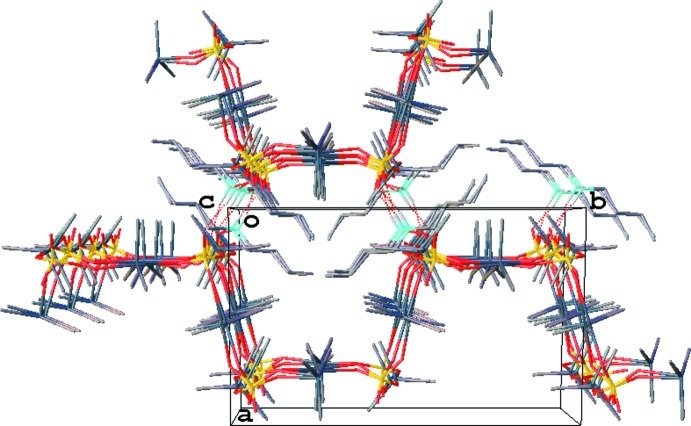
The linking of the sheets through N—H···O hydrogen bonds between the Bu2NH2+ cations and the stannate(IV) sheets (red dotted lines). Only H atoms involved in hydrogen bond interactions are shown for the sake of clarity.

## supplementary crystallographic information

### Comment

Various applications of organotin(IV) compounds explain the focus of research teams, including ours, to the search and characterisation of new organotin(IV) compounds, e.g. Evans & Karpel (1985); Basu *et al.* (2005); Kapoor *et al.* (2005); Samuel *et al.* (2002). In the scope of our research on the coordination ability of oxyanions (Molloy *et al.*, 1989; Diop *et al.*, 2002) and our interest to synthesize new organotin(IV) derivatives for biological tests, we elucidate here the structure of the title compound, (C_8_H_20_N)[(Sn(CH_3_)_3_)_3_(SO_4_)_2_], (I).

Compound (I) has a polymeric structure consisting of three O_2_SnC_3_ moieties, and two different tridentate sulfate ligands (Fig. 1). In the two-dimensional polymeric structure that extends parallel to (100) (Fig. 2) all tin(IV) atoms are five-coordinate, with the trigonal (CH_3_)_3_Sn units axially bridged through sulfate groups. The angles between the apical positions within the trigonal-bipyramidal arrangement indicate a slight deviation from linearity for Sn1 and Sn2 (O1—Sn1—O7 = 172.44 (8)°; O8—Sn2—O2 = 176.59 (11)°) and a considerable deviation for Sn3 (O4—Sn3—O5 = 168.64 (10)°). The Sn—O bonds are in the excepted range [2.262 (2)–2.305 (2) Å] and are shorter than the Sn—O distances in (Bu_4_N)HSO_4_^.^Sn(CH_3_)_3_Cl [2.450 (5)] (Diallo *et al.*, 2009). The dibutylammonium cation connects adjacent anionic layers through N—H···O hydrogen bonding into a three-dimensional network structure (Fig. 3).

### Experimental

(Bu_2_NH_2_)_2_SO_4_^.^H_2_O (*L*) was obtained on mixing a water solution of NH_2_SO_3_H (0.15 g, 1.5 mmol) with Bu_2_NH_2_ (0.78 g, 3 mmol). Hydrolysis of NH_2_SO_3_H in basic media has yielded the sulfate. The title compound has been obtained by reacting (*L*) (0.15 g, 0.4 mmol) with trimethyltin chloride (0.16 g, 0.8 mmol) in ethanol. Slow solvent evaporation yielded colourless crystals. SnMe_3_Cl, the acid NH_2_SO_3_H and Bu_2_NH_2_ were purchased from Aldrich and used without further purification.

### Refinement

Three reflections, (0 1 1), (1 0 0) and (1 1 1), were obstructed by the beam stop and were omitted from the refinement. Disorder is observed for one of the sulfate groups and for the dibutyl ammonium ion. The disorder of the sulfate group extends to the neighboring trimethyl stannyl groups and the occupancy ratio refined to 0.725 (4):0.275 (4). The occupancy ratio for the dibutyl ammonium ion is 0.525 (10):0.475 (10). All equivalent disordered moieties were restrained to have similar geometries (SAME command in *SHELXTL*). Equivalent methyl groups of trimethyl stannyl groups were restrained to have similar ADPs, as were Sn1 and Sn1B and Sn2 and Sn2B. The disordered atoms N1, N1B, C13, C13B, C14 and C14B of the dibutyl ammonium ion were restrained to be approximately isotropic (ISOR 0.005 command in *SHELXTL*). Methyl H atoms were placed onto calculated positions and refined using a riding model, with C—H distances of 0.98 Å and *U*_iso_(H)= 1.5*U*_eq_(C); methylene H atoms were refined with C—H distances of 0.99 Å and *U*_iso_(H)= 1.2*U*_eq_(C); ammonium H atoms were refined with N—H distances of 0.90 Å and *U*_iso_(H)= 1.2*U*_eq_(N).

### Crystal data

**Table:**

(C_8_H_20_N)[Sn_3_(CH_3_)_9_(SO_4_)_2_]	*F*(000) = 1608
*M**_r_* = 813.75	*D*_x_ = 1.693 Mg m^−^^3^
Monoclinic, *P*2_1_/*c*	Mo *K*α radiation, λ = 0.71073 Å
Hall symbol: -P 2ybc	Cell parameters from 9693 reflections
*a* = 11.8847 (2) Å	θ = 2.6–27.5°
*b* = 18.2884 (3) Å	µ = 2.49 mm^−^^1^
*c* = 15.3949 (2) Å	*T* = 173 K
β = 107.380 (1)°	Prism, colourless
*V* = 3193.35 (9) Å^3^	0.06 × 0.04 × 0.04 mm
*Z* = 4	

### Data collection

**Table:**

Bruker SMART CCD diffractometer	7234 independent reflections
Radiation source: fine-focus sealed tube	6265 reflections with *I* > 2σ(*I*)
Graphite monochromator	*R*_int_ = 0.027
phi and ω scans	θ_max_ = 27.5°, θ_min_ = 2.6°
Absorption correction: multi-scan (*SADABS*, Bruker, 2003)	*h* = −15→15
*T*_min_ = 0.865, *T*_max_ = 0.907	*k* = −23→23
69889 measured reflections	*l* = −18→19

### Refinement

**Table:**

Refinement on *F*^2^	Primary atom site location: structure-invariant direct methods
Least-squares matrix: full	Secondary atom site location: difference Fourier map
*R*[*F*^2^ > 2σ(*F*^2^)] = 0.020	Hydrogen site location: inferred from neighbouring sites
*wR*(*F*^2^) = 0.045	H-atom parameters constrained
*S* = 1.06	*w* = 1/[σ^2^(*F*_o_^2^) + (0.0164*P*)^2^ + 2.1021*P*] where *P* = (*F*_o_^2^ + 2*F*_c_^2^)/3
7234 reflections	(Δ/σ)_max_ = 0.004
463 parameters	Δρ_max_ = 0.76 e Å^−^^3^
77 restraints	Δρ_min_ = −0.46 e Å^−^^3^

### Special details

**Table:**

Geometry. All e.s.d.'s (except the e.s.d. in the dihedral angle between two l.s. planes) are estimated using the full covariance matrix. The cell e.s.d.'s are taken into account individually in the estimation of e.s.d.'s in distances, angles and torsion angles; correlations between e.s.d.'s in cell parameters are only used when they are defined by crystal symmetry. An approximate (isotropic) treatment of cell e.s.d.'s is used for estimating e.s.d.'s involving l.s. planes.
Refinement. Refinement of *F*^2^ against ALL reflections. The weighted *R*-factor *wR* and goodness of fit *S* are based on *F*^2^, conventional *R*-factors *R* are based on *F*, with *F* set to zero for negative *F*^2^. The threshold expression of *F*^2^ > σ(*F*^2^) is used only for calculating *R*-factors(gt) *etc*. and is not relevant to the choice of reflections for refinement. *R*-factors based on *F*^2^ are statistically about twice as large as those based on *F*, and *R*- factors based on ALL data will be even larger.

### Fractional atomic coordinates and isotropic or equivalent isotropic displacement parameters (Å^2^)

**Table:**

	*x*	*y*	*z*	*U*_iso_*/*U*_eq_	Occ. (<1)
Sn1	0.19280 (7)	0.52408 (7)	0.43977 (5)	0.02502 (12)	0.725 (4)
C1	0.0484 (7)	0.5964 (5)	0.4208 (8)	0.0473 (16)	0.725 (4)
H1A	0.0467	0.6305	0.3713	0.071*	0.725 (4)
H1B	0.0568	0.6238	0.4770	0.071*	0.725 (4)
H1C	−0.0251	0.5683	0.4052	0.071*	0.725 (4)
C2	0.3636 (5)	0.5703 (3)	0.4693 (8)	0.0441 (16)	0.725 (4)
H2A	0.4138	0.5507	0.5272	0.066*	0.725 (4)
H2B	0.3582	0.6236	0.4739	0.066*	0.725 (4)
H2C	0.3977	0.5581	0.4206	0.066*	0.725 (4)
C3	0.1752 (8)	0.4094 (2)	0.4284 (8)	0.0402 (10)	0.725 (4)
H3A	0.2178	0.3912	0.3872	0.060*	0.725 (4)
H3B	0.0916	0.3966	0.4041	0.060*	0.725 (4)
H3C	0.2080	0.3871	0.4885	0.060*	0.725 (4)
Sn1B	0.1839 (2)	0.54067 (10)	0.43984 (16)	0.02502 (12)	0.275 (4)
C1B	0.0361 (19)	0.6038 (15)	0.436 (2)	0.0473 (16)	0.275 (4)
H1D	0.0052	0.5891	0.4855	0.071*	0.275 (4)
H1E	−0.0247	0.5964	0.3774	0.071*	0.275 (4)
H1F	0.0583	0.6556	0.4426	0.071*	0.275 (4)
C2B	0.3517 (14)	0.5897 (12)	0.467 (2)	0.0441 (16)	0.275 (4)
H2D	0.3535	0.6210	0.4161	0.066*	0.275 (4)
H2E	0.4119	0.5516	0.4760	0.066*	0.275 (4)
H2F	0.3674	0.6193	0.5227	0.066*	0.275 (4)
C3B	0.181 (2)	0.4250 (7)	0.426 (2)	0.0402 (10)	0.275 (4)
H3D	0.1475	0.4120	0.3614	0.060*	0.275 (4)
H3E	0.1326	0.4037	0.4608	0.060*	0.275 (4)
H3F	0.2615	0.4059	0.4484	0.060*	0.275 (4)
Sn2	0.24211 (11)	0.75268 (7)	0.20340 (9)	0.02730 (14)	0.725 (4)
C4	0.3133 (10)	0.7154 (8)	0.1007 (7)	0.0483 (9)	0.725 (4)
H4A	0.3014	0.6625	0.0929	0.072*	0.725 (4)
H4B	0.3979	0.7263	0.1183	0.072*	0.725 (4)
H4C	0.2737	0.7402	0.0432	0.072*	0.725 (4)
C5	0.0582 (5)	0.7575 (10)	0.1795 (8)	0.0491 (14)	0.725 (4)
H5A	0.0396	0.7462	0.2359	0.074*	0.725 (4)
H5B	0.0196	0.7219	0.1325	0.074*	0.725 (4)
H5C	0.0299	0.8068	0.1590	0.074*	0.725 (4)
C6	0.3555 (10)	0.7844 (10)	0.3327 (5)	0.0519 (16)	0.725 (4)
H6A	0.3250	0.7650	0.3806	0.078*	0.725 (4)
H6B	0.3590	0.8379	0.3366	0.078*	0.725 (4)
H6C	0.4347	0.7649	0.3405	0.078*	0.725 (4)
Sn2B	0.2528 (3)	0.7522 (2)	0.1900 (2)	0.02730 (14)	0.275 (4)
C4B	0.323 (3)	0.711 (2)	0.089 (2)	0.0483 (9)	0.275 (4)
H4D	0.3867	0.6771	0.1172	0.072*	0.275 (4)
H4E	0.3529	0.7519	0.0614	0.072*	0.275 (4)
H4F	0.2605	0.6858	0.0428	0.072*	0.275 (4)
C5B	0.0684 (13)	0.759 (3)	0.168 (2)	0.0491 (14)	0.275 (4)
H5D	0.0291	0.7727	0.1050	0.074*	0.275 (4)
H5E	0.0521	0.7964	0.2089	0.074*	0.275 (4)
H5F	0.0387	0.7117	0.1815	0.074*	0.275 (4)
C6B	0.365 (3)	0.784 (3)	0.3199 (15)	0.0519 (16)	0.275 (4)
H6D	0.3242	0.8203	0.3469	0.078*	0.275 (4)
H6E	0.4372	0.8061	0.3129	0.078*	0.275 (4)
H6F	0.3852	0.7414	0.3596	0.078*	0.275 (4)
Sn3	0.48425 (13)	0.49901 (8)	0.23557 (10)	0.02997 (17)	0.725 (4)
C7	0.4615 (6)	0.4916 (5)	0.0949 (4)	0.0587 (14)	0.725 (4)
H7A	0.3880	0.4655	0.0652	0.088*	0.725 (4)
H7B	0.5280	0.4650	0.0848	0.088*	0.725 (4)
H7C	0.4578	0.5409	0.0691	0.088*	0.725 (4)
C8	0.4371 (7)	0.4042 (3)	0.2953 (7)	0.0501 (14)	0.725 (4)
H8A	0.4147	0.4180	0.3494	0.075*	0.725 (4)
H8B	0.5043	0.3706	0.3128	0.075*	0.725 (4)
H8C	0.3703	0.3802	0.2514	0.075*	0.725 (4)
C9	0.5460 (8)	0.5971 (3)	0.3069 (4)	0.0451 (17)	0.725 (4)
H9A	0.5942	0.5855	0.3690	0.068*	0.725 (4)
H9B	0.4786	0.6272	0.3091	0.068*	0.725 (4)
H9C	0.5936	0.6241	0.2755	0.068*	0.725 (4)
Sn3B	0.4842 (3)	0.4875 (2)	0.2556 (2)	0.0272 (4)	0.275 (4)
C7B	0.442 (2)	0.4955 (16)	0.1121 (9)	0.0587 (14)	0.275 (4)
H7D	0.4276	0.4465	0.0853	0.088*	0.275 (4)
H7E	0.5073	0.5184	0.0960	0.088*	0.275 (4)
H7F	0.3705	0.5253	0.0886	0.088*	0.275 (4)
C8B	0.453 (2)	0.3869 (10)	0.306 (2)	0.0501 (14)	0.275 (4)
H8D	0.4458	0.3493	0.2594	0.075*	0.275 (4)
H8E	0.3805	0.3891	0.3234	0.075*	0.275 (4)
H8F	0.5195	0.3746	0.3598	0.075*	0.275 (4)
C9B	0.545 (3)	0.5850 (10)	0.3299 (14)	0.0451 (17)	0.275 (4)
H9D	0.6288	0.5921	0.3355	0.068*	0.275 (4)
H9E	0.5353	0.5810	0.3907	0.068*	0.275 (4)
H9F	0.4996	0.6267	0.2978	0.068*	0.275 (4)
S1	0.22744 (4)	0.56106 (3)	0.66835 (3)	0.02531 (11)	
S2	0.20127 (4)	0.56894 (3)	0.22152 (4)	0.02637 (11)	
O1	0.21738 (14)	0.51080 (8)	0.59177 (10)	0.0346 (3)	
O2	0.25042 (13)	0.63526 (8)	0.63914 (10)	0.0349 (3)	
O3	0.12102 (12)	0.56030 (9)	0.69614 (11)	0.0379 (4)	
O4	0.32836 (12)	0.53761 (8)	0.74657 (10)	0.0336 (3)	
O5	0.29205 (19)	0.53491 (14)	0.1876 (2)	0.0456 (8)	0.725 (4)
O6	0.0959 (3)	0.5803 (2)	0.1412 (3)	0.0323 (8)	0.725 (4)
O7	0.1694 (3)	0.52091 (15)	0.28571 (15)	0.0426 (7)	0.725 (4)
O8	0.2434 (5)	0.6394 (3)	0.2647 (4)	0.0340 (9)	0.725 (4)
O5B	0.2927 (4)	0.5082 (3)	0.2478 (4)	0.0258 (15)	0.275 (4)
O6B	0.1308 (9)	0.5633 (6)	0.1333 (8)	0.035 (2)	0.275 (4)
O7B	0.1313 (4)	0.5615 (4)	0.2883 (4)	0.0275 (14)	0.275 (4)
O8B	0.2728 (12)	0.6375 (8)	0.2442 (10)	0.037 (3)	0.275 (4)
C10	0.1466 (18)	0.2765 (11)	0.122 (2)	0.118 (10)	0.525 (10)
H10A	0.2085	0.3102	0.1168	0.177*	0.525 (10)
H10B	0.1496	0.2318	0.0872	0.177*	0.525 (10)
H10C	0.1590	0.2641	0.1858	0.177*	0.525 (10)
C11	0.0283 (16)	0.3123 (10)	0.0838 (13)	0.078 (6)	0.525 (10)
H11A	−0.0334	0.2802	0.0944	0.093*	0.525 (10)
H11B	0.0113	0.3187	0.0173	0.093*	0.525 (10)
C12	0.0245 (14)	0.3855 (8)	0.1273 (15)	0.055 (5)	0.525 (10)
H12A	0.0433	0.3789	0.1940	0.066*	0.525 (10)
H12B	0.0858	0.4175	0.1160	0.066*	0.525 (10)
C13	−0.0918 (10)	0.4225 (7)	0.0928 (10)	0.046 (3)	0.525 (10)
H13A	−0.1550	0.3893	0.0987	0.056*	0.525 (10)
H13B	−0.1076	0.4347	0.0277	0.056*	0.525 (10)
N1	−0.0914 (11)	0.4906 (6)	0.1463 (10)	0.035 (2)	0.525 (10)
H1G	−0.0931	0.4777	0.2037	0.042*	0.525 (10)
H1H	−0.0238	0.5172	0.1525	0.042*	0.525 (10)
C14	−0.1959 (10)	0.5347 (5)	0.0970 (8)	0.046 (2)	0.525 (10)
H14A	−0.2682	0.5058	0.0911	0.055*	0.525 (10)
H14B	−0.1920	0.5454	0.0350	0.055*	0.525 (10)
C15	−0.2040 (12)	0.6053 (6)	0.1442 (8)	0.052 (3)	0.525 (10)
H15A	−0.1275	0.6311	0.1577	0.062*	0.525 (10)
H15B	−0.2187	0.5945	0.2029	0.062*	0.525 (10)
C16	−0.3018 (10)	0.6557 (5)	0.0880 (8)	0.082 (3)	0.525 (10)
H16A	−0.2873	0.6660	0.0291	0.098*	0.525 (10)
H16B	−0.3782	0.6298	0.0748	0.098*	0.525 (10)
C17	−0.3111 (7)	0.7268 (4)	0.1333 (8)	0.091 (3)	0.525 (10)
H17A	−0.3710	0.7575	0.0917	0.136*	0.525 (10)
H17B	−0.2348	0.7519	0.1495	0.136*	0.525 (10)
H17C	−0.3337	0.7176	0.1886	0.136*	0.525 (10)
C10B	0.1311 (16)	0.2763 (11)	0.118 (2)	0.089 (8)	0.475 (10)
H10D	0.2031	0.3041	0.1223	0.134*	0.475 (10)
H10E	0.1269	0.2341	0.0776	0.134*	0.475 (10)
H10F	0.1322	0.2593	0.1783	0.134*	0.475 (10)
C11B	0.0255 (19)	0.3243 (11)	0.0792 (15)	0.075 (6)	0.475 (10)
H11C	−0.0467	0.2946	0.0703	0.090*	0.475 (10)
H11D	0.0264	0.3421	0.0187	0.090*	0.475 (10)
C12B	0.0191 (12)	0.3895 (9)	0.1379 (17)	0.049 (5)	0.475 (10)
H12C	0.0920	0.4189	0.1487	0.059*	0.475 (10)
H12D	0.0142	0.3723	0.1976	0.059*	0.475 (10)
C13B	−0.0881 (12)	0.4379 (7)	0.0930 (13)	0.055 (4)	0.475 (10)
H13C	−0.0844	0.4524	0.0320	0.066*	0.475 (10)
H13D	−0.1603	0.4082	0.0842	0.066*	0.475 (10)
N1B	−0.0993 (13)	0.5058 (7)	0.1445 (11)	0.036 (2)	0.475 (10)
H1I	−0.1010	0.4929	0.2019	0.043*	0.475 (10)
H1J	−0.0317	0.5324	0.1507	0.043*	0.475 (10)
C14B	−0.2004 (10)	0.5555 (6)	0.1088 (8)	0.043 (2)	0.475 (10)
H14C	−0.2746	0.5277	0.0987	0.052*	0.475 (10)
H14D	−0.1985	0.5753	0.0494	0.052*	0.475 (10)
C15B	−0.1988 (14)	0.6180 (8)	0.1730 (8)	0.045 (2)	0.475 (10)
H15C	−0.1276	0.6483	0.1803	0.054*	0.475 (10)
H15D	−0.1970	0.5988	0.2336	0.054*	0.475 (10)
C16B	−0.3104 (8)	0.6649 (6)	0.1338 (8)	0.069 (3)	0.475 (10)
H16C	−0.3800	0.6322	0.1174	0.082*	0.475 (10)
H16D	−0.3188	0.6985	0.1819	0.082*	0.475 (10)
C17B	−0.3107 (9)	0.7085 (6)	0.0530 (7)	0.104 (4)	0.475 (10)
H17D	−0.2393	0.7386	0.0671	0.155*	0.475 (10)
H17E	−0.3803	0.7401	0.0360	0.155*	0.475 (10)
H17F	−0.3125	0.6756	0.0023	0.155*	0.475 (10)

### Atomic displacement parameters (Å^2^)

**Table:**

	*U*^11^	*U*^22^	*U*^33^	*U*^12^	*U*^13^	*U*^23^
Sn1	0.02974 (15)	0.0191 (3)	0.02525 (9)	−0.0002 (2)	0.00679 (9)	0.0016 (2)
C1	0.040 (2)	0.039 (2)	0.054 (4)	0.0089 (15)	0.0003 (19)	−0.003 (2)
C2	0.0365 (18)	0.062 (4)	0.0331 (15)	−0.011 (2)	0.0091 (15)	0.002 (3)
C3	0.0535 (16)	0.027 (2)	0.0405 (15)	−0.004 (2)	0.0147 (16)	−0.012 (3)
Sn1B	0.02974 (15)	0.0191 (3)	0.02525 (9)	−0.0002 (2)	0.00679 (9)	0.0016 (2)
C1B	0.040 (2)	0.039 (2)	0.054 (4)	0.0089 (15)	0.0003 (19)	−0.003 (2)
C2B	0.0365 (18)	0.062 (4)	0.0331 (15)	−0.011 (2)	0.0091 (15)	0.002 (3)
C3B	0.0535 (16)	0.027 (2)	0.0405 (15)	−0.004 (2)	0.0147 (16)	−0.012 (3)
Sn2	0.0307 (2)	0.02653 (8)	0.0250 (4)	−0.00035 (13)	0.00894 (16)	0.0003 (2)
C4	0.058 (3)	0.042 (3)	0.055 (3)	0.0052 (16)	0.0309 (17)	−0.008 (2)
C5	0.0383 (17)	0.040 (2)	0.074 (3)	−0.0031 (19)	0.0245 (17)	0.005 (3)
C6	0.072 (3)	0.0378 (16)	0.033 (3)	−0.005 (2)	−0.004 (2)	0.002 (3)
Sn2B	0.0307 (2)	0.02653 (8)	0.0250 (4)	−0.00035 (13)	0.00894 (16)	0.0003 (2)
C4B	0.058 (3)	0.042 (3)	0.055 (3)	0.0052 (16)	0.0309 (17)	−0.008 (2)
C5B	0.0383 (17)	0.040 (2)	0.074 (3)	−0.0031 (19)	0.0245 (17)	0.005 (3)
C6B	0.072 (3)	0.0378 (16)	0.033 (3)	−0.005 (2)	−0.004 (2)	0.002 (3)
Sn3	0.02517 (15)	0.0352 (4)	0.0292 (5)	0.0003 (2)	0.0076 (3)	−0.0033 (3)
C7	0.045 (3)	0.096 (3)	0.032 (3)	0.000 (2)	0.0078 (19)	−0.003 (3)
C8	0.041 (3)	0.056 (4)	0.052 (3)	−0.009 (3)	0.011 (2)	0.004 (3)
C9	0.0316 (13)	0.044 (3)	0.061 (4)	−0.005 (2)	0.016 (3)	−0.016 (3)
Sn3B	0.0278 (4)	0.0324 (9)	0.0210 (10)	0.0030 (5)	0.0068 (6)	−0.0003 (6)
C7B	0.045 (3)	0.096 (3)	0.032 (3)	0.000 (2)	0.0078 (19)	−0.003 (3)
C8B	0.041 (3)	0.056 (4)	0.052 (3)	−0.009 (3)	0.011 (2)	0.004 (3)
C9B	0.0316 (13)	0.044 (3)	0.061 (4)	−0.005 (2)	0.016 (3)	−0.016 (3)
S1	0.0256 (2)	0.0280 (3)	0.0235 (3)	−0.00012 (19)	0.0090 (2)	−0.0016 (2)
S2	0.0255 (2)	0.0269 (3)	0.0264 (3)	−0.00036 (19)	0.0073 (2)	0.0025 (2)
O1	0.0516 (9)	0.0286 (8)	0.0245 (8)	−0.0029 (7)	0.0130 (7)	−0.0031 (6)
O2	0.0423 (8)	0.0288 (8)	0.0358 (9)	−0.0019 (6)	0.0148 (7)	−0.0030 (7)
O3	0.0281 (7)	0.0505 (10)	0.0378 (9)	0.0058 (7)	0.0143 (7)	0.0074 (8)
O4	0.0269 (7)	0.0415 (9)	0.0307 (9)	0.0042 (6)	0.0061 (6)	−0.0021 (7)
O5	0.0299 (12)	0.0513 (16)	0.0538 (19)	0.0044 (10)	0.0098 (11)	−0.0087 (14)
O6	0.0282 (18)	0.035 (2)	0.0298 (16)	0.0027 (13)	0.0022 (13)	0.0038 (13)
O7	0.0663 (19)	0.0372 (15)	0.0227 (13)	−0.0174 (14)	0.0108 (12)	0.0007 (11)
O8	0.044 (2)	0.0272 (14)	0.028 (2)	−0.0040 (15)	0.0069 (14)	0.0019 (13)
O5B	0.021 (2)	0.022 (3)	0.035 (4)	0.000 (2)	0.009 (2)	0.000 (2)
O6B	0.037 (6)	0.039 (6)	0.023 (4)	0.003 (4)	0.001 (4)	0.005 (3)
O7B	0.019 (3)	0.041 (4)	0.023 (3)	0.006 (2)	0.008 (2)	−0.002 (3)
O8B	0.042 (6)	0.030 (4)	0.032 (7)	−0.003 (4)	0.001 (4)	0.008 (4)
C10	0.127 (15)	0.070 (12)	0.16 (2)	0.020 (10)	0.040 (13)	−0.037 (12)
C11	0.092 (12)	0.070 (8)	0.085 (13)	−0.036 (7)	0.047 (10)	−0.043 (8)
C12	0.077 (9)	0.051 (8)	0.038 (7)	−0.007 (6)	0.019 (6)	−0.005 (5)
C13	0.056 (4)	0.055 (4)	0.029 (4)	−0.024 (3)	0.013 (3)	−0.015 (3)
N1	0.031 (3)	0.038 (4)	0.029 (3)	−0.006 (2)	0.000 (2)	0.004 (3)
C14	0.049 (3)	0.045 (4)	0.041 (4)	−0.002 (3)	0.009 (3)	0.000 (3)
C15	0.041 (3)	0.047 (6)	0.061 (8)	−0.014 (4)	0.008 (5)	−0.015 (5)
C16	0.073 (5)	0.081 (6)	0.093 (8)	0.008 (4)	0.028 (6)	0.006 (6)
C17	0.082 (5)	0.047 (5)	0.142 (10)	−0.001 (3)	0.031 (5)	0.006 (5)
C10B	0.069 (8)	0.092 (15)	0.108 (15)	−0.037 (9)	0.029 (8)	−0.039 (11)
C11B	0.095 (13)	0.053 (7)	0.061 (10)	−0.010 (7)	−0.001 (7)	−0.004 (7)
C12B	0.053 (7)	0.058 (9)	0.041 (7)	−0.027 (6)	0.020 (5)	−0.024 (5)
C13B	0.062 (5)	0.058 (5)	0.044 (5)	−0.023 (4)	0.013 (3)	−0.006 (4)
N1B	0.038 (4)	0.041 (4)	0.031 (4)	−0.005 (3)	0.013 (3)	0.006 (3)
C14B	0.035 (3)	0.049 (5)	0.038 (4)	0.004 (3)	0.001 (3)	0.004 (4)
C15B	0.044 (4)	0.039 (5)	0.047 (6)	−0.002 (4)	0.005 (4)	−0.008 (4)
C16B	0.044 (4)	0.065 (7)	0.095 (8)	0.006 (3)	0.019 (5)	0.014 (6)
C17B	0.134 (9)	0.063 (6)	0.104 (8)	0.016 (5)	0.020 (6)	0.020 (6)

### Geometric parameters (Å, º)

**Table:**

Sn1—C1	2.117 (5)	C8B—H8F	0.9800
Sn1—C2	2.119 (5)	C9B—H9D	0.9800
Sn1—C3	2.110 (4)	C9B—H9E	0.9800
Sn1—O1	2.2829 (17)	C9B—H9F	0.9800
Sn1—O7	2.305 (2)	S1—O3	1.4513 (15)
Sn1B—C1B	2.088 (13)	S1—O1	1.4714 (15)
Sn1B—C2B	2.111 (13)	S1—O2	1.4803 (15)
Sn1B—C3B	2.126 (13)	S1—O4	1.4866 (15)
Sn1B—O1	2.317 (3)	S2—O6B	1.370 (11)
Sn1B—O7B	2.260 (6)	S2—O7	1.455 (2)
Sn2—C4	2.117 (5)	S2—O8	1.468 (5)
Sn2—C5	2.107 (5)	S2—O5	1.470 (2)
Sn2—C6	2.124 (5)	S2—O6	1.489 (4)
Sn2—O2^i^	2.2906 (19)	S2—O8B	1.496 (13)
Sn2—O8	2.274 (6)	S2—O7B	1.510 (5)
Sn2B—C4B	2.100 (14)	S2—O5B	1.522 (5)
Sn2B—C5B	2.120 (14)	O2—Sn2B^iii^	2.200 (4)
Sn2B—C6B	2.128 (14)	O2—Sn2^iii^	2.2906 (19)
Sn2B—O2^i^	2.200 (4)	O4—Sn3^ii^	2.262 (2)
Sn2B—O8B	2.243 (15)	O4—Sn3B^ii^	2.285 (4)
Sn3—C7	2.106 (5)	C10—C11	1.502 (12)
Sn3—C8	2.115 (5)	C10—H10A	0.9800
Sn3—C9	2.117 (5)	C10—H10B	0.9800
Sn3—O4^ii^	2.262 (2)	C10—H10C	0.9800
Sn3—O5	2.277 (3)	C11—C12	1.506 (11)
Sn3B—C7B	2.119 (13)	C11—H11A	0.9900
Sn3B—C8B	2.074 (13)	C11—H11B	0.9900
Sn3B—C9B	2.125 (13)	C12—C13	1.487 (11)
Sn3B—O4^ii^	2.285 (4)	C12—H12A	0.9900
Sn3B—O5B	2.274 (6)	C12—H12B	0.9900
C1—H1A	0.9800	C13—N1	1.491 (10)
C1—H1B	0.9800	C13—H13A	0.9900
C1—H1C	0.9800	C13—H13B	0.9900
C2—H2A	0.9800	N1—C14	1.486 (10)
C2—H2B	0.9800	N1—H1G	0.9206
C2—H2C	0.9800	N1—H1H	0.9195
C3—H3A	0.9800	N1—H1I	0.8986
C3—H3B	0.9800	N1—H1J	1.0317
C3—H3C	0.9800	C14—C15	1.498 (9)
C1B—H1D	0.9800	C14—H14A	0.9900
C1B—H1E	0.9800	C14—H14B	0.9900
C1B—H1F	0.9800	C15—C16	1.533 (10)
C2B—H2D	0.9800	C15—H15A	0.9900
C2B—H2E	0.9800	C15—H15B	0.9900
C2B—H2F	0.9800	C16—C17	1.496 (11)
C3B—H3D	0.9800	C16—H16A	0.9900
C3B—H3E	0.9800	C16—H16B	0.9900
C3B—H3F	0.9800	C17—H17A	0.9800
C4—H4A	0.9800	C17—H17B	0.9800
C4—H4B	0.9800	C17—H17C	0.9800
C4—H4C	0.9800	C10B—C11B	1.501 (13)
C5—H5A	0.9800	C10B—H10D	0.9800
C5—H5B	0.9800	C10B—H10E	0.9800
C5—H5C	0.9800	C10B—H10F	0.9800
C6—H6A	0.9800	C11B—C12B	1.511 (13)
C6—H6B	0.9800	C11B—H11C	0.9900
C6—H6C	0.9800	C11B—H11D	0.9900
C4B—H4D	0.9800	C12B—C13B	1.535 (12)
C4B—H4E	0.9800	C12B—H12C	0.9900
C4B—H4F	0.9800	C12B—H12D	0.9900
C5B—H5D	0.9800	C13B—N1B	1.502 (11)
C5B—H5E	0.9800	C13B—H13C	0.9900
C5B—H5F	0.9800	C13B—H13D	0.9900
C6B—H6D	0.9800	N1B—C14B	1.475 (10)
C6B—H6E	0.9800	N1B—H1G	1.0296
C6B—H6F	0.9800	N1B—H1H	0.8937
C7—H7A	0.9800	N1B—H1I	0.9207
C7—H7B	0.9800	N1B—H1J	0.9195
C7—H7C	0.9800	C14B—C15B	1.507 (10)
C8—H8A	0.9800	C14B—H14C	0.9900
C8—H8B	0.9800	C14B—H14D	0.9900
C8—H8C	0.9800	C15B—C16B	1.542 (10)
C9—H9A	0.9800	C15B—H15C	0.9900
C9—H9B	0.9800	C15B—H15D	0.9900
C9—H9C	0.9800	C16B—C17B	1.477 (11)
C7B—H7D	0.9800	C16B—H16C	0.9900
C7B—H7E	0.9800	C16B—H16D	0.9900
C7B—H7F	0.9800	C17B—H17D	0.9800
C8B—H8D	0.9800	C17B—H17E	0.9800
C8B—H8E	0.9800	C17B—H17F	0.9800
			
C3—Sn1—C1	123.6 (4)	O7—S2—O8	110.2 (2)
C3—Sn1—C2	118.7 (3)	O7—S2—O5	110.70 (17)
C1—Sn1—C2	117.7 (3)	O8—S2—O5	110.19 (18)
C3—Sn1—O1	87.6 (3)	O7—S2—O6	108.9 (2)
C1—Sn1—O1	93.3 (3)	O8—S2—O6	110.0 (3)
C2—Sn1—O1	90.1 (3)	O5—S2—O6	106.81 (17)
C3—Sn1—O7	84.9 (3)	O6B—S2—O8B	115.5 (8)
C1—Sn1—O7	91.7 (3)	O6B—S2—O7B	111.7 (5)
C2—Sn1—O7	92.6 (3)	O8B—S2—O7B	107.9 (5)
O1—Sn1—O7	172.44 (8)	O6B—S2—O5B	112.7 (4)
C1B—Sn1B—C2B	120.5 (10)	O8B—S2—O5B	103.8 (6)
C1B—Sn1B—C3B	124.0 (11)	O7B—S2—O5B	104.4 (3)
C2B—Sn1B—C3B	115.4 (9)	S1—O1—Sn1	135.22 (9)
C1B—Sn1B—O7B	84.5 (10)	S1—O1—Sn1B	127.57 (10)
C2B—Sn1B—O7B	95.1 (9)	S1—O2—Sn2B^iii^	137.17 (12)
C3B—Sn1B—O7B	94.1 (9)	S1—O2—Sn2^iii^	130.78 (9)
C1B—Sn1B—O1	92.9 (10)	S1—O4—Sn3^ii^	135.28 (10)
C2B—Sn1B—O1	91.7 (9)	S1—O4—Sn3B^ii^	126.92 (12)
C3B—Sn1B—O1	82.0 (9)	S2—O5—Sn3	141.14 (17)
O7B—Sn1B—O1	173.07 (18)	S2—O7—Sn1	135.08 (14)
Sn1B—C1B—H1D	109.5	S2—O8—Sn2	130.8 (3)
Sn1B—C1B—H1E	109.5	S2—O5B—Sn3B	139.4 (3)
H1D—C1B—H1E	109.5	S2—O7B—Sn1B	132.8 (3)
Sn1B—C1B—H1F	109.5	S2—O8B—Sn2B	135.0 (9)
H1D—C1B—H1F	109.5	C10—C11—C12	111.4 (13)
H1E—C1B—H1F	109.5	C10—C11—H11A	109.4
Sn1B—C2B—H2D	109.5	C12—C11—H11A	109.4
Sn1B—C2B—H2E	109.5	C10—C11—H11B	109.4
H2D—C2B—H2E	109.5	C12—C11—H11B	109.4
Sn1B—C2B—H2F	109.5	H11A—C11—H11B	108.0
H2D—C2B—H2F	109.5	C13—C12—C11	113.2 (12)
H2E—C2B—H2F	109.5	C13—C12—H12A	108.9
Sn1B—C3B—H3D	109.5	C11—C12—H12A	108.9
Sn1B—C3B—H3E	109.5	C13—C12—H12B	108.9
H3D—C3B—H3E	109.5	C11—C12—H12B	108.9
Sn1B—C3B—H3F	109.5	H12A—C12—H12B	107.7
H3D—C3B—H3F	109.5	C12—C13—N1	109.4 (9)
H3E—C3B—H3F	109.5	C12—C13—H13A	109.8
C5—Sn2—C4	120.6 (4)	N1—C13—H13A	109.8
C5—Sn2—C6	119.1 (4)	C12—C13—H13B	109.8
C4—Sn2—C6	120.3 (4)	N1—C13—H13B	109.8
C5—Sn2—O8	89.4 (5)	H13A—C13—H13B	108.2
C4—Sn2—O8	93.6 (4)	C14—N1—C13	107.9 (8)
C6—Sn2—O8	86.3 (5)	C14—N1—H1G	112.0
C5—Sn2—O2^i^	93.6 (5)	C13—N1—H1G	108.5
C4—Sn2—O2^i^	83.5 (4)	C14—N1—H1H	109.7
C6—Sn2—O2^i^	93.6 (5)	C13—N1—H1H	111.0
O8—Sn2—O2^i^	176.59 (11)	H1G—N1—H1H	107.8
C4B—Sn2B—C5B	121.5 (12)	N1—C14—C15	112.7 (8)
C4B—Sn2B—C6B	121.1 (12)	N1—C14—H14A	109.0
C5B—Sn2B—C6B	117.3 (12)	C15—C14—H14A	109.0
C4B—Sn2B—O2^i^	92.1 (12)	N1—C14—H14B	109.0
C5B—Sn2B—O2^i^	89.1 (14)	C15—C14—H14B	109.0
C6B—Sn2B—O2^i^	90.8 (14)	H14A—C14—H14B	107.8
C4B—Sn2B—O8B	85.4 (13)	C14—C15—C16	113.1 (8)
C5B—Sn2B—O8B	96.0 (14)	C14—C15—H15A	109.0
C6B—Sn2B—O8B	86.7 (15)	C16—C15—H15A	109.0
O2^i^—Sn2B—O8B	174.8 (4)	C14—C15—H15B	109.0
Sn2B—C4B—H4D	109.5	C16—C15—H15B	109.0
Sn2B—C4B—H4E	109.5	H15A—C15—H15B	107.8
H4D—C4B—H4E	109.5	C17—C16—C15	114.1 (9)
Sn2B—C4B—H4F	109.5	C17—C16—H16A	108.7
H4D—C4B—H4F	109.5	C15—C16—H16A	108.7
H4E—C4B—H4F	109.5	C17—C16—H16B	108.7
Sn2B—C5B—H5D	109.5	C15—C16—H16B	108.7
Sn2B—C5B—H5E	109.5	H16A—C16—H16B	107.6
H5D—C5B—H5E	109.5	C11B—C10B—H10D	109.5
Sn2B—C5B—H5F	109.5	C11B—C10B—H10E	109.5
H5D—C5B—H5F	109.5	H10D—C10B—H10E	109.5
H5E—C5B—H5F	109.5	C11B—C10B—H10F	109.5
Sn2B—C6B—H6D	109.5	H10D—C10B—H10F	109.5
Sn2B—C6B—H6E	109.5	H10E—C10B—H10F	109.5
H6D—C6B—H6E	109.5	C10B—C11B—C12B	114.3 (13)
Sn2B—C6B—H6F	109.5	C10B—C11B—H11C	108.7
H6D—C6B—H6F	109.5	C12B—C11B—H11C	108.7
H6E—C6B—H6F	109.5	C10B—C11B—H11D	108.7
C7—Sn3—C8	115.2 (4)	C12B—C11B—H11D	108.7
C7—Sn3—C9	120.4 (3)	H11C—C11B—H11D	107.6
C8—Sn3—C9	124.4 (3)	C11B—C12B—C13B	111.6 (13)
C7—Sn3—O4^ii^	85.7 (2)	C11B—C12B—H12C	109.3
C8—Sn3—O4^ii^	94.8 (2)	C13B—C12B—H12C	109.3
C9—Sn3—O4^ii^	90.7 (3)	C11B—C12B—H12D	109.3
C7—Sn3—O5	82.9 (2)	C13B—C12B—H12D	109.3
C8—Sn3—O5	90.4 (2)	H12C—C12B—H12D	108.0
C9—Sn3—O5	94.7 (3)	N1B—C13B—C12B	115.6 (11)
O4^ii^—Sn3—O5	168.64 (10)	N1B—C13B—H13C	108.4
C8B—Sn3B—C7B	116.0 (11)	C12B—C13B—H13C	108.4
C8B—Sn3B—C9B	127.8 (10)	N1B—C13B—H13D	108.4
C7B—Sn3B—C9B	116.1 (9)	C12B—C13B—H13D	108.4
C8B—Sn3B—O5B	83.2 (8)	H13C—C13B—H13D	107.4
C7B—Sn3B—O5B	90.6 (6)	C14B—N1B—C13B	120.3 (10)
C9B—Sn3B—O5B	93.7 (8)	C14B—N1B—H1I	105.4
C8B—Sn3B—O4^ii^	96.2 (8)	C13B—N1B—H1I	109.0
C7B—Sn3B—O4^ii^	85.9 (6)	C14B—N1B—H1J	107.5
C9B—Sn3B—O4^ii^	90.0 (8)	C13B—N1B—H1J	106.3
O5B—Sn3B—O4^ii^	175.8 (2)	H1I—N1B—H1J	107.8
Sn3B—C7B—H7D	109.5	N1B—C14B—C15B	112.0 (9)
Sn3B—C7B—H7E	109.5	N1B—C14B—H14C	109.2
H7D—C7B—H7E	109.5	C15B—C14B—H14C	109.2
Sn3B—C7B—H7F	109.5	N1B—C14B—H14D	109.2
H7D—C7B—H7F	109.5	C15B—C14B—H14D	109.2
H7E—C7B—H7F	109.5	H14C—C14B—H14D	107.9
Sn3B—C8B—H8D	109.5	C14B—C15B—C16B	108.9 (9)
Sn3B—C8B—H8E	109.5	C14B—C15B—H15C	109.9
H8D—C8B—H8E	109.5	C16B—C15B—H15C	109.9
Sn3B—C8B—H8F	109.5	C14B—C15B—H15D	109.9
H8D—C8B—H8F	109.5	C16B—C15B—H15D	109.9
H8E—C8B—H8F	109.5	H15C—C15B—H15D	108.3
Sn3B—C9B—H9D	109.5	C17B—C16B—C15B	114.5 (10)
Sn3B—C9B—H9E	109.5	C17B—C16B—H16C	108.6
H9D—C9B—H9E	109.5	C15B—C16B—H16C	108.6
Sn3B—C9B—H9F	109.5	C17B—C16B—H16D	108.6
H9D—C9B—H9F	109.5	C15B—C16B—H16D	108.6
H9E—C9B—H9F	109.5	H16C—C16B—H16D	107.6
O3—S1—O1	111.15 (10)	C16B—C17B—H17D	109.5
O3—S1—O2	110.76 (9)	C16B—C17B—H17E	109.5
O1—S1—O2	107.90 (9)	H17D—C17B—H17E	109.5
O3—S1—O4	108.77 (9)	C16B—C17B—H17F	109.5
O1—S1—O4	108.76 (9)	H17D—C17B—H17F	109.5
O2—S1—O4	109.45 (9)	H17E—C17B—H17F	109.5
			
O3—S1—O1—Sn1	106.91 (14)	O7—S2—O8—Sn2	−153.4 (3)
O2—S1—O1—Sn1	−14.72 (16)	O5—S2—O8—Sn2	84.2 (4)
O4—S1—O1—Sn1	−133.36 (13)	O6—S2—O8—Sn2	−33.3 (4)
O3—S1—O1—Sn1B	103.90 (14)	O8B—S2—O8—Sn2	63 (2)
O2—S1—O1—Sn1B	−17.73 (16)	O7B—S2—O8—Sn2	−127.2 (4)
O4—S1—O1—Sn1B	−136.37 (13)	O5B—S2—O8—Sn2	128.2 (4)
C3—Sn1—O1—S1	−173.5 (3)	C5—Sn2—O8—S2	72.3 (5)
C1—Sn1—O1—S1	−49.9 (3)	C4—Sn2—O8—S2	−48.3 (5)
C2—Sn1—O1—S1	67.8 (2)	C6—Sn2—O8—S2	−168.5 (5)
C3—Sn1—O1—Sn1B	−156.0 (6)	O6B—S2—O5B—Sn3B	95.0 (7)
C1—Sn1—O1—Sn1B	−32.5 (6)	O7—S2—O5B—Sn3B	−154.8 (6)
C2—Sn1—O1—Sn1B	85.3 (6)	O8—S2—O5B—Sn3B	−50.1 (6)
C1B—Sn1B—O1—S1	−52.2 (9)	O5—S2—O5B—Sn3B	43.9 (4)
C2B—Sn1B—O1—S1	68.5 (6)	O6—S2—O5B—Sn3B	106.0 (5)
C3B—Sn1B—O1—S1	−176.1 (8)	O8B—S2—O5B—Sn3B	−30.6 (7)
C1B—Sn1B—O1—Sn1	143.3 (10)	O7B—S2—O5B—Sn3B	−143.5 (5)
C2B—Sn1B—O1—Sn1	−96.1 (8)	C8B—Sn3B—O5B—S2	177.8 (10)
C3B—Sn1B—O1—Sn1	19.3 (9)	C7B—Sn3B—O5B—S2	−66.1 (10)
O3—S1—O2—Sn2B^iii^	46.6 (2)	C9B—Sn3B—O5B—S2	50.2 (8)
O1—S1—O2—Sn2B^iii^	168.50 (19)	O6B—S2—O7B—Sn1B	164.8 (7)
O4—S1—O2—Sn2B^iii^	−73.3 (2)	O7—S2—O7B—Sn1B	62.0 (5)
O3—S1—O2—Sn2^iii^	44.60 (15)	O8—S2—O7B—Sn1B	−70.9 (5)
O1—S1—O2—Sn2^iii^	166.47 (11)	O5—S2—O7B—Sn1B	51.2 (8)
O4—S1—O2—Sn2^iii^	−75.33 (13)	O6—S2—O7B—Sn1B	179.2 (5)
O3—S1—O4—Sn3^ii^	165.74 (13)	O8B—S2—O7B—Sn1B	−67.2 (8)
O1—S1—O4—Sn3^ii^	44.55 (16)	O5B—S2—O7B—Sn1B	42.8 (6)
O2—S1—O4—Sn3^ii^	−73.12 (15)	C1B—Sn1B—O7B—S2	148.8 (10)
O3—S1—O4—Sn3B^ii^	171.41 (16)	C2B—Sn1B—O7B—S2	28.6 (8)
O1—S1—O4—Sn3B^ii^	50.21 (18)	C3B—Sn1B—O7B—S2	−87.4 (10)
O2—S1—O4—Sn3B^ii^	−67.45 (17)	O6B—S2—O8B—Sn2B	27.1 (11)
O6B—S2—O5—Sn3	178.2 (6)	O7—S2—O8B—Sn2B	−131.5 (7)
O7—S2—O5—Sn3	−67.0 (3)	O8—S2—O8B—Sn2B	−88 (2)
O8—S2—O5—Sn3	55.3 (4)	O5—S2—O8B—Sn2B	111.9 (8)
O6—S2—O5—Sn3	174.7 (3)	O6—S2—O8B—Sn2B	3.6 (10)
O8B—S2—O5—Sn3	62.7 (6)	O7B—S2—O8B—Sn2B	−98.7 (9)
O7B—S2—O5—Sn3	−60.5 (5)	O5B—S2—O8B—Sn2B	151.0 (8)
O5B—S2—O5—Sn3	−47.9 (3)	C4B—Sn2B—O8B—S2	−82.5 (13)
C7—Sn3—O5—S2	−166.7 (4)	C5B—Sn2B—O8B—S2	38.8 (13)
C8—Sn3—O5—S2	78.0 (4)	C6B—Sn2B—O8B—S2	155.9 (13)
C9—Sn3—O5—S2	−46.6 (3)	C10—C11—C12—C13	−179 (2)
O4^ii^—Sn3—O5—S2	−164.7 (4)	C11—C12—C13—N1	174.2 (18)
O6B—S2—O7—Sn1	−153.6 (5)	C12—C13—N1—C14	166.7 (16)
O8—S2—O7—Sn1	−10.0 (4)	C13—N1—C14—C15	−179.4 (13)
O5—S2—O7—Sn1	112.2 (3)	N1—C14—C15—C16	172.4 (13)
O6—S2—O7—Sn1	−130.7 (3)	C14—C15—C16—C17	−179.6 (11)
O8B—S2—O7—Sn1	5.2 (7)	C10B—C11B—C12B—C13B	178 (2)
O7B—S2—O7—Sn1	−61.2 (4)	C11B—C12B—C13B—N1B	−178 (2)
O5B—S2—O7—Sn1	99.2 (3)	C12B—C13B—N1B—C14B	−178.6 (18)
C3—Sn1—O7—S2	−156.8 (4)	C13B—N1B—C14B—C15B	176.8 (16)
C1—Sn1—O7—S2	79.6 (4)	N1B—C14B—C15B—C16B	−176.8 (13)
C2—Sn1—O7—S2	−38.2 (3)	C14B—C15B—C16B—C17B	−71.4 (16)
O6B—S2—O8—Sn2	−13.0 (6)		

Symmetry codes: (i) *x*, −*y*+3/2, *z*−1/2; (ii) −*x*+1, −*y*+1, −*z*+1; (iii) *x*, −*y*+3/2, *z*+1/2.

### Hydrogen-bond geometry (Å, º)

**Table:**

*D*—H···*A*	*D*—H	H···*A*	*D*···*A*	*D*—H···*A*
N1—H1*H*···O6	0.92	1.88	2.785 (16)	168
N1*B*—H1*J*···O6*B*	0.92	2.11	2.98 (2)	159
